# Supplementary material for: Machine Learning Coronary Artery Disease Prediction Based on Imaging and Non-Imaging Data
Source: Diagnostics (Basel). 2022 Jun 14;12(6):1466. doi: 10.3390/diagnostics12061466 (PMC9221964; doi:10.3390/diagnostics12061466)
Supplement: Supplementary file 1 [file diagnostics-12-01466-s001.zip › diagnostics-1706701-supplementary.pdf]

**Supplementary Materials**

$$F^*(x) = \arg min\ E_{y,x}L(y, F(x)) = \arg\min E_x\left[\left(E_y\left(L(y, F(x))\right)\Big|x\right)\right]. \quad (S1)$$

$$F(x; \{\beta_m, a_m\}_1^M) = \sum_{m=1}^{M} \beta_m h(x; a_m), \quad (S2)$$

where $h(x; a)$ is a simple parameterized function of the input variables $x$, characterized by the parameters $a = \{a_1, a_2, \ldots\ldots\}$, whereas $\{\beta_m, a_m\}_1^M$ denotes the entire parameter set [1].

The Gradient Boosting classification algorithm [2] implements a numerical implementation, minimizing Eq. S1 and yields an additive expansion of the form:

$$F^*(x) = \sum_{m=0}^{M} f_m(x), \quad (S3)$$

where $f_0$ is an initial guess and $\{f_m\}_1^M$ are successive "boosts", each based on the sequence of preceding steps. More specifically, for the steepest-descent

$$f_m(x) = -p_m g_m(x), \quad (S4)$$

with

$$g_m(x) = \left[\frac{\partial E_y\left[\left(L(y, F(x))|x\right)\right]}{\partial F(x)}\right]_{F(x)=F_{m-1}(x)}, \quad (S5)$$

$$F_{m-1}(x) = \sum_{i=0}^{m-1} f_i(x), \quad (S6)$$

and

$$p_m = argmin_p E_{y,x} L(y, F_{m-1}(x) - p g_m(x)), \quad (S7)$$

Subsequently, based on the training dataset $D = \{(x_i, Y_i)\}_{i=1}^N$ and aiming to minimize Eq. S1, we try a "greedy-stagewise" approach to obtain

$$(\beta_m, a_m) = argmin_{\beta,a} \sum_{i=1}^{N} L(y_i, F_{m-1}(x_i) + \beta h(x_i; a)), \quad (S8)$$

and then

$$F_m(x) = F_{m-1}(x) + \beta_m h(x; a_m). \quad (S9)$$

Given an approximation of $F_{m-1}(x)$, the function $\beta_m h(x; a_m)$ can be considered as the best greedy step towards the data based estimate of $F^*(x)$, whereas the data based analogue of the unconstrained negative gradient;

$$-g_m(x_i) = -\left[\frac{\partial L(y_i, F(x_i))}{\partial F(x_i)}\right]_{F(x)=F_{m-1}(x)}, \quad (S10)$$

gives the best steepest-descent step direction $-g_m = \{-g_m(x_i)\}_1^N$ in the N-dimensional data space at $F_{m-1}(x)$. The most highly correlated $h(x; a)$ with $-g_m(x)$ over the data distribution can be obtained from the solution

$$\alpha_m = argmin_{a,\beta} \sum_{i=1}^{N} [-g_m(x_i) - \beta h(x_i; a)]^2. \quad (S11)$$

The line search is performed by

$$p_m = argmin_p \sum_{i=1}^{N} L\left(y_i, F_{m-1}(x_i) + p h(x_i; a_m)\right), \quad (S12)$$

and the final approximation is given by:

$$F_m(x) = F_{m-1}(x) + p_m h(x; a_m). \quad (S13)$$

**Table S1**: SVM RFE Algorithm [3].

| |
|---|
| **Input:** |
| **Training Examples**: $X_0 = [x_1, x_2, \ldots, x_k, \ldots. x_l]^T$ |
| Class labels: $y = [y_1, y_2, \ldots, y_k, \ldots. y_l]^T$ |
| **Initialize:** |

**Subset of features**: $s = [1,2, \ldots, d]$

**Feature ranked list** $r = [\ \ ]$

**Repeat until** $s = [\ \ ]$

**Restrict training examples to good feature indices:** $X = X_0(:, s)$

**Train the classifier:** $a = SVM\_train(X, y)$

Minimize over $a_k$: $J = \frac{1}{2}\sum_{hk} y_h y_k a_h a_k (x_h \cdot x_k + \lambda\delta_{hk}) - \sum_k a_k$, subject to: $0 \leq a_k \leq C$ and $\sum_k a_k y_k = 0$

Outputs: Parameters: $a_k$

**Compute the weight vector of dimension length(s):** $w = \sum_k a_k y_k x_k$

**Compute the ranking criteria**: $c_i = (w_i)^2$, for all $i$

**Find the feature with smallest ranking criterion**: $f = argmin(c)$

**Update feature ranked list:** $r = [s(f), r]$

**Eliminate the feature with smallest ranking criterion**: $s = s(1{:}f-1, f+1{:}length(s))$

**Output**: Feature ranked list $r$

*CTCA image analysis and Three-dimensional reconstruction*

The utilized geometrical features were extracted based on published studies, which can accurately reconstruct the coronary artery anatomy, by providing 3D models of the lumen, the outer wall and two different types of atherosclerotic plaque, the calcified (CP) and the noncalcified plaques (NCP). Briefly, the proposed methodology is summarized in seven steps: (a) the preprocessing step in which the Frangi vesselness filter is implemented, (b) the blooming effect removal step implementing a deconvolution technique, (c) the coronary vessel centerline extraction implementing a minimum cost path based approach, (d) the estimation of the lumen, outer wall and CP intensity weight functions, (e) the segmentation of the lumen, outer wall and CP implementing an active contour based model, (f) the NCP segmentation using a dynamic thresholding technique and (g) the 3D surface construction based on Marching cubes 8 approach.

This methodology is integrated in a dedicated software tool, which semi-automatically can provide the detailed 3D coronary artery anatomy [4,5].

*Calculation of the SmartFFR index*

In order to calculate SmartFFR, blood flow finite element simulations are carried out on the reconstructed 3D models of the coronary arteries. The arterial lumen is discretized into tetrahedral finite elements of face size that ranges from 0.09 to 0.12 mm and the respective Navier-Stokes and continuity equations are then solved using Finite elements. A transient blood flow simulation is performed on the 3D reconstructed artery. The flow is considered laminar and the blood is treated as a Newtonian fluid with density 1050 kg/m$^3$ and dynamic viscosity 0.0035 Pa·s. For each timestep, the Pd/Pa value is calculated in order to construct the Pd/Pa vs. flow curve. The calculated Pd/Pa values for every timestep are then connected to create the appropriate patient-specific curve. The patient-specific curve is constructed for a flow range of 0-4 ml/s and the SmartFFR value is calculated by dividing the area under the patient specific curve to the respective area under the curve of the respective healthy arterial segment [6].

*Medical centers*

The medical centers which provide the utilized imaging data are shown in Table 2, below.

**Table S2:** Medical centres provided imaging data and the final utilized study population.

| Centre | Total of CTCA data for each center |
|---|---|
| FTGM (Pisa) | 87 |
| UTU (Turku) | 54 |
| UZH (Zurich) | 31 |
| Barcelona | 32 |
| Warsaw | 38 |
| Naples | 1 |
| Viareggio | 20 |

**Full list of eligibility, inclusion, exclusion and exit criteria**

**Eligibility criteria:**

A. Clinical history and lifestyle data records available at one-time point.

B. At least one previous CCTA examination performed for suspected CHD and of good quality to allow for:

a) Non-invasive FFR-CT assessment

b) Quantitative (automated) 17 segments (AHA) analysis and measurement with ≤10% error of MLA (mm2), lumen area stenosis (%), mean plaque burden ($mm^3$

), plaque burden at MLA (%), and remodeling index,

c) Plaque phenotype assessment: HU based classification in calcified, non-calcified (LAP) and mixed, napkin-ring sign, CAC score.

C. Previous blood and plasma sample available for retrospective analysis

**Inclusion criteria:**

1) male and female subjects

2) aged 45-82 years

3) Caucasian population

4) submitted to CCTA for suspected CHD between 2009 and 2012 (in the context of EVINCI and ARTreat FPVII studies) at the Hospitals reported in "SMARTool Clinical Center" document and satisfying the elegibility criteria reported above

5) submitted to clinical Follow-up in the last 6 months with stable clinical conditions and documented CHD or persistent intermediate/high probability of CHD

6) Signed informed consents (clinical and genetic)

**Exclusion criteria:**

1) Multi-vessel severe disease (3 vessels and/or LM disease with >90% stenosis).

2) Severe coronary calcification (CAC score > 600).

3) Having undergone surgical procedures related to heart diseases (valve replacement, CRT or CRTD treatment, any surgery of the heart or arteries).

4) Documented MACE at history (myocardial infarction, severe heart failure, recurrent angina) in the last 6 months with/without revascularization

5) Documented severe peripheral vascular disease (carotid, femoral)

6) Surgery of carotid and/or peripheral arteries or cerebral ischemic attack

7) History/surgery of Abdominal Aortic Aneurysm(AAA).

8) Severe Heart failure (NYHA Class III-IV)

9) LV dysfunction (left ventricle EF <40%).

10) Atrial fibrillation.

11) Lack of written informed consent (clinical consent and/or genetic consent)

12) Pregnancy (evaluated by urine test) and breastfeeding

13) Active Cancer

14) Asthma

15) Cardiomyopathy or congenital heart disease

16) Significant valvular disease (hemodynamically significant valvular stenosis or insufficiency by echoDoppler)

17) Renal dysfunction (creatinine > 1.3 mg/dL)

18) Chronic Kidney Disease (eGFR < 30 ml/min/1.73 m2)

19) Hepatic failure (at least 3 of the following: albumin < 3.5 g/dL; prolonged prothrombin time–PT; jaundice; ascites)

20) Waldenstrom disease

21) Multiple myeloma

22) Autoimmune/Acute inflammatory disease

23) Previous severe adverse reaction to iodine contrast agent

24) Positivity at blood tests for HIV, Hepatitis B and C (CRF number 1-clinical evaluation)

**Exit Criteria:**

A) Informed consent retired by the patient (genetic or clinical)

B) Adverse events to contrast medium during

**References**


1. Robert, C. Machine learning, a probabilistic perspective. **2014**.
2. Friedman, J.H. Greedy Function Approximation: A Gradient Boosting Machine. *The Annals of Statistics* **2001**, *29*, 1189-1232.
3. Guyon, I.; Weston, J.; Barnhill, S.; Vapnik, V. Gene selection for cancer classification using support vector machines. *Machine learning* **2002**, *46*, 389-422.
4. Kigka, V.I.; Rigas, G.; Sakellarios, A.; Siogkas, P.; Andrikos, I.O.; Exarchos, T.P.; Loggitsi, D.; Anagnostopoulos, C.D.; Michalis, L.K.; Neglia, D.; et al. 3D reconstruction of coronary arteries and atherosclerotic plaques based on computed tomography angiography images. *Biomedical Signal Processing and Control* **2018**, *40*, 286-294, doi:https://doi.org/10.1016/j.bspc.2017.09.009.
5. Kigka, V.I.; Sakellarios, A.; Kyriakidis, S.; Rigas, G.; Athanasiou, L.; Siogkas, P.; Tsompou, P.; Loggitsi, D.; Benz, D.C.; Buechel, R. A three-dimensional quantification of calcified and non-calcified plaques in coronary arteries based on computed tomography coronary angiography images: Comparison with expert's annotations and virtual histology intravascular ultrasound. *Computers in biology and medicine* **2019**, *113*, 103409.
6. Papafaklis, M.I.; Muramatsu, T.; Ishibashi, Y.; Lakkas, L.S.; Nakatani, S.; Bourantas, C.V.; Ligthart, J.; Onuma, Y.; Echavarria-Pinto, M.; Tsirka, G. Fast virtual functional assessment of intermediate coronary lesions using routine angiographic data and blood flow simulation in humans: comparison with pressure wire-fractional flow reserve. *EuroIntervention: journal of EuroPCR in collaboration with the Working Group on Interventional Cardiology of the European Society of Cardiology* **2014**, *10*, 574-583.
